# Assessment of Pregnant Women’s Satisfaction with Model of Care Initiative: Antenatal Care Service at Primary Health Care in Cluster One in Riyadh, Saudi Arabia

**DOI:** 10.3390/healthcare10010151

**Published:** 2022-01-13

**Authors:** Saad M. Alhaqbani, Amen A. Bawazir

**Affiliations:** Riyadh Health Cluster One, Riyadh 11623, Saudi Arabia; bawazira@ksau-hs.edu.sa

**Keywords:** antenatal care, satisfaction, primary health care center, educational degree, pregnant

## Abstract

The current study assessed pregnant women’s satisfaction with antenatal care (ANC) services at primary health care centers (PHCs) in Riyadh Cluster One. The study was conducted at 11 PHCs where the ANC initiative has been implemented. A total of 646 pregnant women were enrolled. A questionnaire was completed by participants to measure the level of satisfaction with the provided services, care, and consultation. Subsequently, the data were analyzed to determine the significant differences and conduct regression analysis. The overall satisfaction with initial triage assessment, provided services, consultation, and examination was 93.7%, 87.8%, 71.8%, and 53.9%, respectively. Regarding ANC services, education was the only statistically significant variable that influenced patient satisfaction (*p* < 0.05). In contrast, satisfaction with the provided care was significantly related to all the variables studied. For consultation, education (*p* < 0.001) and monthly income (*p* < 0.05) were the statistically significant role players. In the regression analysis, secondary education was statistically significantly related to the provided services, consultation, and examination. Despite the satisfactory level of ANC at the selected PHCs, higher patient satisfaction could be achieved in the future by improving the consultation and examination practices. Overall satisfaction with the health care workers at PHCs is high. Incorporating implied ameliorations would enhance the quality of services and patient satisfaction.

## 1. Introduction

Antenatal care (ANC), or prenatal care, is one of the most critical and essential services that must be provided to women during pregnancy across the globe, either in hospitals or through primary health care centers (PHCs). ANC starts from the first day of the confirmation of pregnancy and continues until delivery. The World Health Organization (WHO) and most international guidelines insist that pregnant women should complete at least four visits to a health care facility during pregnancy [1]. 

Patient satisfaction is one of the main factors that aids in evaluating and elevating health care services. Therefore, countries are considering the opinion of pregnant women as an essential component of ANC improvement programs. This step is taken to ensure the patient’s continuous follow-ups and warrant a good relationship between a pregnant woman and the physician [2,3]. Satisfaction of the patients is an important parameter for evaluating the PHCs’ performance [4]. 

According to the recent report by the Ministry of Health of Saudi Arabia, the total number of planned PHCs in 2021 was 2900. ANC services are being provided in some of the PHCs [5]. ANC services at the PHCs facilitate part of prenatal care and serves as a bridge between the primary, secondary, and tertiary levels of health care through the early identification of any risk associated with the pregnancy [6].

As part of Saudi Vision 2030, the kingdom has been divided into different health cluster zones, and each zone is responsible for the health care services to its overall population. A total of eight health clusters are already running out of 20 planned across the kingdom. Three clusters are in operation in Riyadh city. The ANC initiative is being implemented in 20 out of 43 PHCs [7]. The aim of this initiative, which could be replicated in other locations worldwide as well, was to cater to all medium- and low-risk pregnant women at PHCs. This initiative was accompanied by adequate infrastructure improvements and the training of physicians and nurses.

Since the establishment of these health clusters, a general systematic evaluation is being undertaken. Therefore, this research was planned to assess pregnant women’s satisfaction with the ANC initiative as a new concept in PHCs belonging to Riyadh Cluster One. The study identified the provided services for pregnant women and focused on assessing satisfaction levels and determining the compliance of routine antenatal visits.

## 2. Materials and Methods

### 2.1. Study Site and Design

The study was conducted at 11 PHCs where the ANC initiative has been implemented in Riyadh Cluster One. The advanced health care facility at the study site is King Saudi Medical City, whereas the secondary health facilities include King Salman Hospital, Aliman Hospital, and Imam Abdulrahman Alfaisal Hospital. Saudi women under 18 and non-Saudi women were excluded. This research used a cross-sectional survey design. A self-administrative questionnaire (Supplementary File S1) was distributed to all pregnant women who visited primary health care facilities for ANC. The study was conducted in collaboration with the community health excellence in Cluster One in Riyadh from August to September 2020.

### 2.2. Sample Size

We used the Raosoft sample size calculation method for estimating the required sample size through the following equation:

*n* = [(Z0.95)^2^ × *p* × (1 − *p*)]/(0.05)^2^

where *n* is the sample size, Z is the Z value (1.96 for 95% CI), and *p* is the proportion or prevalence that met our criteria; *p* was set as 0.5, because the proportion was not known. 

The calculated sample size was 384 to achieve a confidence level of 95% and a precision of ±0.05. However, because cluster and stratified sampling were employed, we used 1.5 as a design effect [8], resulting in an increase in the sample size by 192 cases (384 × 1.5 = 576 participants), in addition to the expected non-respondent cases of 8% (70 participants) [9]. Therefore, a total of 646 participants were enrolled in the study (384 + 192 + 70).

### 2.3. Sampling Technique

Using proportionate stratified sampling based on the rate of attendance in each PHC in 2019, we calculated the pregnant visitors to ANC at selected PHCs as 1207 and estimated the sample size required (384). By using a design effect, the total sample size was 646 participants.

### 2.4. Data Collection Methods, Instructions Used, and Measurements

A validated English-version questionnaire used elsewhere was adopted [1]. The questionnaire was translated into Arabic. This questionnaire was piloted on 30 women (5%, who were not included in the study) to test the reliability and internal consistency. Accordingly, a few amendments were incorporated. The reliability test showed a Cronbach’s alpha of 0.838 for the questions related to satisfaction on received advice, and 0.858 for the questions related to the satisfaction of the level of care.

The main variables in our questionnaire included sociodemographic variables (age, education level, monthly income, number of previous pregnancies, and PHC visits). The data for patients’ weight, height, and blood pressure were recorded. Moreover, blood tests, ultrasound, urine tests, and physical examination results were also logged. Furthermore, it was also considered whether the patient was taking an iron or folic acid prescription or any other medication. Regarding patients’ preferences about the ANC services, their feedback was collected for waiting time, satisfaction about services, comfortability of the clinic, privacy, and qualification of the team. A trained nurse was assigned in each center to help in tracking pregnant women’s enrollment and to ensure that the pregnant women filled in the questionnaires before leaving. 

### 2.5. Data Management and Analysis Plan

Revision of the questionnaire content and data completeness was performed. Moreover, data entry was double-checked by two persons who transformed paper data to digital data to ensure quality and accuracy. The statistical analysis was conducted using IBM SPSS Statistics for Windows, version 25.0 (IBM Corp., Armonk, NY, USA). Fisher’s exact tests and Pearson’s chi-squared analyses were performed for categorical variables. The Shapiro–Wilk tests was used to test the normality assumptions. Results are expressed as the number (%), mean standard deviation (SD), or median (min–max). *p*-values < 0.05 were considered statistically significant. 

To determine satisfaction with the services, the mean values were set as the cutoff point to assess satisfaction among the pregnant patients for different parameters. Then, the findings were recorded as binary variables by considering the mean value as adequate satisfaction, and hence given the number 1. In contrast, those below the mean were considered inadequate and given the number 0. Moreover, to gain a deeper insight into the influence of different variables on women’s satisfaction with provided services, provided care, and provided consultation, simple linear regression analyses of the data were also performed using modeling regression, which revealed that education played a major role in satisfaction.

### 2.6. Ethical Considerations

Ethical approval for the study was obtained from the institutional review board. Our questionnaire was anonymous. Moreover, the aim of this study was shared with the contributors before involving them. Furthermore, the participants were given the right to withdraw from the study at any time. Additionally, informed consent was obtained from each participant. Both hard and soft copies of the data were saved within the Ministry of National Guard Health Affairs (MNGHA) premises under the license number SP20/169/R.

## 3. Results

### 3.1. Sociodemographic Characteristics of the Participants

The sociodemographic characteristics of the 646 pregnant women that constituted the study population are presented in Supplementary Table S1. The data indicated considerable variations in the demographic parameters of the participants. The average monthly income was SAR 6500 (Saudi riyals; SD ± 6114) (approximately USD 1733; exchange rate: USD 1 = SAR 3.75). The mean age of the participants was 30.5 years (SD ± 6.7). The level of education of most of the women in the study was secondary (46.7%), followed by university (35.4%) and basic education (17.8%). 

### 3.2. Overall Adequate Satisfaction among Participants

A summary of the overall adequate satisfaction of different variables is presented in Table 1. A high level of satisfaction was observed among pregnant women for initial triage assessment (93.7%). The results reflected the overall adequacy of services in PHCs. In detail, 96.6% of the enrolled pregnant women were weighed, the height of 97.1% of women was measured, and blood pressure was recorded in 95% of the participants. 

For patient investigation and physical examination, urine and blood samples were collected in 69.5% and 69.7% of participants, respectively. Moreover, ultrasounds of 62.7% of the pregnant women were performed. Furthermore, stomach palpations were performed on 71.5% of the respondents, whereas uterine height was measured in the case of 60.1% of the entrants. Regarding medication, it was noticed that 86.8% of the respondents received iron pills, folic acid, or iron with folic acid, and 85.0% of the women took other medicines dispensed to them. The overall adequate satisfaction for the investigation, physical examination, and medication was 53.9% (intermediate satisfaction).

Women were counseled for any danger signs expected during pregnancy (86.4%) and diet medications (85.3%). Moreover, counseling was also conducted regarding family planning and delivery (in 79.1% and 71.8% patients, respectively). Overall, most participants agreed that the health care workers’ behavior and the services provided were excellent (satisfaction over 90%). Furthermore, the pregnant women trusted the staff regarding privacy (95.2%), their health care decisions (94.4%), and time devoted to consulting (94.1%) and explaining (94.0%). 

### 3.3. Satisfaction with Provided Services

Pregnant women aged between 26 and 35 years indicated higher satisfaction with the provided services (54.5%) than the pregnant women from other age categories (*p*-value 0.501; Table 2). Among all the variables dissected in the study for satisfaction with the provided services, education was the only one that showed statistical significance (*p*-value 0.03). The pregnant women with a secondary education had higher satisfaction with ANC services with a percentage of 48.7%, followed by those who had a university degree (34.0%).

### 3.4. Satisfaction with Provided Care

Regarding participants’ characteristics and satisfaction with the provided care, the variables of age, education, monthly income, number of pregnancies, and PHC visits were all significantly associated (Table 2). The pregnant women aged between 26 and 35 years showed maximum satisfaction (59.5%). Overall, the *p*-value for this variable was 0.003.

In addition, education was statistically associated with the level of satisfaction with provided care (*p*-value 0.000). Pregnant women holding a secondary degree had higher satisfaction (56.0%), indicating that education played a paramount role in women’s satisfaction. In addition, pregnant women with a monthly income of SAR 5000–7000 had the highest satisfaction with the provided care (*p*-value = 0.001). The entrants with 2–3 pregnancies seemed to be more highly satisfied with the provided care (57.8%) than any other category. Moreover, the women who visited the health care centers more than once reported higher satisfaction with the provided care (70.4%; *p*-value = 0.001).

### 3.5. Satisfaction with Provided Consultation

Regarding the consultations provided (Table 2), education (*p*-value = 0.000) and monthly income (*p*-value = 0.045) were significantly associated, whereas other variables were statistically non-significant. Parallel to observations for other variables, the pregnant women holding a secondary education exhibited higher satisfaction (51.9%). Moreover, the financial status also seemed to be a paramount factor related to consultation satisfaction (*p*-value 0.045). For this variable, the middle-income group (SAR 5000–7000) demonstrated the highest association with consultation satisfaction (42.5%).

The pregnant women aged between 26 and 35 years illustrated the highest satisfaction with the consultation (55.8%). Moreover, women with 2–3 pregnancies indicated high satisfaction with the consultation provided (53.7%). With reference to prior visits to the PHCs, a non-significant association with the provided consultation was noticed.

### 3.6. Regression Analysis of Satisfaction about Provided Services and Participants Characteristics

To gain a deeper insight into the influence of various variables on women’s satisfaction with ANC services, we conducted a regression analysis of the data. The results of this analysis to assess the impact of the variables (age, education, monthly income, number of pregnancies, and visits to the PHC) on the satisfaction about provided services are presented in Table 3. Women who had a secondary education level (AOR: 1.503; 95% CI: 1.038–2.175) were more satisfied with the provided care compared with those who had completed university education.

### 3.7. Regression Analysis of Satisfaction about Provided Care and Participants Characteristics

Regression analysis of satisfaction about the provided care showed that many of the variables significantly impacted the patient satisfaction (Table 3). The AOR values for basic education, 2–3 pregnancies, and first visit to PHC were 0.365, 2.449, and 0.471, respectively, with a *p*-value of 0.000 for each. Moreover, the characteristics such as secondary education, monthly income SAR ≤ 4900, monthly income SAR 5000–7000, and one prior pregnancy illustrated AORs of 1.503, 1.456, 1.618, and 2.019, respectively (*p*-values 0.031, 0.090, 0.025, and 0.004, correspondingly).

### 3.8. Regression Analysis of Satisfaction about Provided Consultation and Participant Characteristics

Parallel to the regression analysis for provided services, secondary education appeared to be the most influential variable for consultation (AOR 1.899 and *p*-value 0.002). The AOR and *p*-value for patients with basic education were 0.734 and 0.202, respectively. The impacts of other variables, including the number of pregnancies and monthly income, were not statistically significant regarding satisfaction of consultation. 

## 4. Discussion

The current study addressed questions regarding the feasibility of new ANC services to pregnant women at PHCs. Women’s satisfaction with the provided facilities indicates the efficacy of the Cluster One system and may decide the future direction of such projects. To the best of our knowledge, this is the first study assessing the satisfaction level among pregnant women of the recently implemented model of care in the Cluster One area in Riyadh as part of the health care transformation. Our study showed that the average level of satisfaction with ANC at the PHCs was 76.8%. This proportion of satisfaction was higher than that found in similar studies in Oman (59%), southern Ethiopia (32%), and Uganda (40%) [10].

We noticed the least satisfaction among patients in the lowest educational category. This finding suggested that education level plays an important role in women’s satisfaction with the services provided. Half of our participants had fewer than four children, and many of them attended hospitals during their first or second pregnancy because the service would have not been well established in PHCs at the time. However, now, with services available at PHCs, they started visiting the health care facility from day one; overall, the entrants revisited the PHCs in 60.8% of cases. The percentage of revisiting was slightly higher than what was observed in Egypt [11], indicating that women were more content with the ANC services. 

The preliminary assessment of the patients included weight, height, and blood pressure, because these are important parameters to measure during any pregnancy, either at the first visit or during follow-ups [12]. These evaluations can also help in assessing the need for urgency in treatment, facilitating the triage system, and referrals to an appropriate consultant, as observed in South Africa [13]. The overall satisfaction of 93.7% for initial triage assessment showed the promising performance of PHCs, Ministry of Health’s National Standards for ANC, and the use of mother and child passports [14].

In the current study, almost half of the participants underwent testing such as blood and urine sampling and ultrasound, which is relatively lower than mentioned by other researchers elsewhere [15]; for instance, in neighboring Baghdad, Iraq [16]. These results suggested room for improvement, because these tests and ultrasound investigations should be included regularly as supportive procedures to determine early indications of any risk to the mother’s or baby’s health [17]. 

The uterine height was measured in half of the participants, which is lower than that reported by [18] in Nigeria. Overall, 71.8% of the pregnant women were satisfied with the physicians’ counseling. The percentage of satisfaction was lower than expected, because more than 1 year had passed since the full implementation of the ANC initiative. Nevertheless, patient satisfaction was significantly higher compared with that observed in Egypt [19]. Counseling about future pregnancies is also one of the main aspects of ANC services [20]. In this study, more than half of the pregnant women were recommended to use family planning, similar to another report from Nigeria [21]. 

Almost 80% of our participants were advised to exclusively breastfeed at least in the first few months, in line with the recommendations of the WHO and UNICEF [22]. In the third trimester, an arrangement should be ensured between the PHC and the hospital where the baby will be delivered. In concordance with some previous studies, this arrangement was made by the PHCs for most of the participants [23,24]. 

The satisfaction with the facility and staff was above 90%. The participants agreed that they were treated with respect, and the staff had sufficient medical information and knowledge. Moreover, satisfaction about the accessibility of PHCs, availability of medication, and patients’ privacy was also promising. These observations were similar to the previous studies from Ethiopia [25], Nigeria [23], and Kazakhstan [24].

Most participants enrolled in this study planned to keep attending PHCs until their delivery schedule. Therefore, the current study presents evidence about the promising role of PHCs in providing pregnancy services and highlights the room for improvement and future research for certain variables. Incorporating implied ameliorations in the PHC operations may further enhance patient satisfaction and the quality of services. 

## 5. Limitations

This study used a cross-sectional design, was conducted in Cluster One only, and excluded non-Saudi women, which may limit the general applicability of this research. For a broader outlook, data should be collected from various clusters and all PHCs. Moreover, the study duration was limited to one month due to the COVID-19 pandemic, which resulted in reduced visits. Furthermore, the lack of human resources was also a limitation of this study. 

## 6. Conclusions

Only those with a secondary education had a high level of satisfaction with ANC. The results indicated that there is a dire need to form public communication teams whose role should be to announce and promote antenatal care in primary health care centers to both educators and non-educators. Low integration between physicians and patients was observed; therefore, physicians should receive training on how to deal with a pregnant woman in a proper and professional manner. It is also important to assign a professional health educator to each PHC to guide pregnant women through the steps of family planning after birth.

## Figures and Tables

**Table 1:** Overall adequate satisfaction of participants.

Items	No.	%
Overall adequate satisfaction of initial triage assessment	605	93.7
Overall adequate satisfaction of investigation, physical examination, and medication	348	53.9
Overall adequate consultation	464	71.8
Overall adequate satisfaction for HCWs and provided services	567	87.8

**Table 2:** Level of satisfaction with provided services, provided care, and provided consultation.

Variables	Categories	Provided Services					Provided Care					Provided Consultation				
		Inadequate		Adequate			Inadequate		Adequate			Inadequate		Adequate		
		No.	%	No.	%	*p*-Value	No.	%	No.	%	*p*-Value	No.	%	No.	%	*p*-Value
Age groups (years)	≤25 years	23	29.1	138	24.3	0.501	75	25.2	86	24.7	0.003	48	26.4	113	24.4	0.546
	26–35 years	43	54.4	309	54.5		145	48.7	207	59.5		93	51.1	259	55.8	
	≥36 years	13	16.5	120	21.2		78	26.2	55	15.8		41	22.5	92	19.8	
Education	Basic	17	21.5	98	17.3	0.030	84	28.2	31	8.9	<0.000	47	25.8	68	14.7	<0.000
	Secondary	26	32.9	276	48.7		107	35.9	195	56.0		61	33.5	241	51.9	
	University	36	45.6	193	34.0		107	35.9	122	35.1		74	40.7	155	33.4	
Monthly income	SAR ≤ 4900	19	24.1	196	34.6	0.072	95	31.9	120	34.5	0.001	67	36.8	148	31.9	0.045
	SAR 5000–7000	31	39.2	224	39.5		102	34.2	153	44.0		58	31.9	197	42.5	
	SAR > 7000	29	36.7	147	25.9		101	33.9	75	21.6		57	31.3	119	25.6	
Number of pregnancies	One pregnancy	23	29.1	152	26.8	0.598	79	26.5	96	27.6	0.000	55	30.2	120	25.9	0.058
	2–3 pregnancies	42	53.2	286	50.4		127	42.6	201	57.8		79	43.4	249	53.7	
	≥4 pregnancies	14	17.7	129	22.8		92	30.9	51	14.7		48	26.4	95	20.5	
First visit to the PHC	Yes	30	38.0	223	39.3	0.817	150	50.3	103	29.6	0.000	82	45.1	171	36.9	0.055
	No	49	62.0	344	60.7		148	49.7	245	70.4		100	54.9	293	63.1	

**Table 3:** Regression analysis between the satisfaction level with provided services, provided care, provided consultation, and participants characteristics.

Variables	Categories	*n* Adequate	Not Adequate	UAOR	95% CI	AOR	95% CI
Provided services							
Age groups (years)	≤25 years	138	23	0.751	0.327–1.727	-	-
	26–35 years	309	43	0.865	0.421–1.778	-	-
	≥36 years	120	13	Reference	-		
Education	Basic	98	17		0.483–1.758	1.075	0.575–2.011
	Secondary	276	26		1.041–3.136	1.980	1.157–3.387
	University	193	36	Reference	-	-	-
Monthly income	SAR ≤ 4900	196	19		1.035–3.666	-	-
	SAR 5000–7000	224	31		0.775–2.395	-	-
	SAR > 7000	147	29	Reference	-	-	-
Number of Pregnancies	One pregnancy	152	23	0.725	0.321–1.636	-	-
	2–3 pregnancies	286	42	0.731	0.355–1.502	-	-
	≥4 pregnancies	129	14	Reference	-	-	-
First visit to the PHC	Yes	223	30		0.708–1.939	-	-
	No	344	49	Reference	-	-	-
Provided care							
Age groups (years)	≤25 years	86	75	1.126	0.641–1.979	-	-
	26–35 years	207	145	1.338	0.830–2.155	-	-
	≥36 years	55	78	Reference	-	-	-
Education	Basic	31	84	0.372	0.224–0.617	0.365	0.220–0.605
	Secondary	195	107	1.521	1.049–2.205	1.503	1.038–2.175
	University	122	107	Reference	-	Reference	-
Monthly income	SAR ≤ 4900	120	95	1.448	0.938–2.236	1.456	0.943–2.247
	SAR 5000–7000	153	102	1.610	1.056–2.455	1.618	1.061–2.466
	SAR > 7000	75	101	Reference	-	Reference	-
Number of Pregnancies	One pregnancy	96	79	1.892	1.099–3.257	2.019	1.245–3.271
	2–3 pregnancies	201	127	2.238	1.391–3.600	2.449	1.589–3.776
	≥4 pregnancies	51	92	Reference	-	Reference	-
First visit to the PHC	Yes	103	150	0.477	0.338–0.675	0.471	0.334–0.665
	No	245	148	Reference	-	Reference	-
Provided consultation							
Age groups (years)	≤25 years	113	48	0.969	0.534–1.757	-	-
	26–35 years	259	93	1.052	0.640–1.728	-	-
	≥36 years	92	41	Reference	-	-	-
Education	Basic	68	47	0.747	0.464–1.204	0.734	0.456–1.180
	Secondary	241	61	1.885	1.258–2.825	1.899	1.269–2.842
	University	155	74	Reference	-	Reference	-
Monthly income	SAR ≤ 4900	148	67	0.902	0.578–1.407	0.911	0.584–1.421
	SAR 5000–7000	197	58	1.422	0.910–2.221	1.457	0.935–2.270
	SAR > 7000	119	57	Reference	-	Reference	-
Number of Pregnancies	One pregnancy	120	55	1.006	0.574–1.762	0.972	0.597–1.585
	2–3 pregnancies	249	79	1.490	0.909–2.442	1.506	0.965–2.352
	≥4 pregnancies	95	48	Reference	-	Reference	-
First visit to the PHC	yes	171	82	0.838	0.583–1.205	-	-
	no	293	100	Reference	-	-	-

*n*, number of participants; UAOR, unadjusted odds ratio; AOR, adjusted odds ratio; CI, confidence interval.

## Data Availability

Data and detailed results can be requested by email to: haqbanism@gmail.com.

## Supplementary Materials

The following supporting information can be downloaded at: https://www.mdpi.com/article/10.3390/healthcare10010151/s1. Table S1: The sociodemographic characteristics of the participants (n = 646); File S1: Assessment of pregnant women satisfaction on antenatal care services in primary health care.

## Abbreviations

ANC	Antenatal care
PHCs	Primary health care centers
HCWs	Health care workers
SAR	Saudi riyal
