# Supplementary material for: Assessment of Pregnant Women’s Satisfaction with Model of Care Initiative: Antenatal Care Service at Primary Health Care in Cluster One in Riyadh, Saudi Arabia
Source: Healthcare (Basel). 2022 Jan 13;10(1):151. doi: 10.3390/healthcare10010151 (PMC8775455; doi:10.3390/healthcare10010151)
Supplement: Supplementary file 1 [file healthcare-10-00151-s001.zip › healthcare-1511944-Supplementary File S1.pdf]

RYD-20-419812-72211

King Abdullah International Medical Research Center
KAIMRC
APPROVED
IRB
Institutional Review Board

Kingdom of Saudi Arabia

King Saud bin Abdulaziz University for Health Sciences

College of Public Health and Health Informatics

Public Health

Assessment of pregnant women satisfaction on antenatal care services in primary health care

Questionnaire

The purpose of the survey is “to *assess pregnant women satisfaction with antenatal care services in central first cluster primary health care in Riyadh, KSA”*.

Your participation is completely voluntary, and your responses will be completely anonymous. The data that will be collected will be analyzed at the group level only. You do not have to answer any question you’d rather not answer. There are no consequences if you decide not to complete the survey.

If you agree to complete the survey, no need to write your name on it if you feel that is not convenient to you.

The university has approved this project.

Thank you for your participation.

(1) **IDENTIFICATION AND DEMOGRAPHIC CHARACTERISTICS** Record Response

1.01	Patient MRN		
1.02	Patient Age (years)		
	1.03 Residency area		
	1.04 Monthly income		
	1.05 Number of pregnancies		
1.06	What is the highest education you ever attended	Pre-primary	0
		Primary	1
		Secondary	2
		Higher	3
1.07	What is your marital status	Single	1
		Married/	2
		Widowed	3
		Divorced/separated	4
1.08	Is this your first antenatal visit at this facility for this pregnancy	Yes	1
		No	2

(2) **TRIAGE & TREATMENT**

2.01	During this visit, were you weighed?	Yes	1
		No	2
2.02	During this visit, was your height measured?	Yes	1
		No	2
2.03	During this visit, did someone measure your	Yes	1

	blood pressure?	No	2
2.04	During this visit, did you give a urine sample?	Yes	1
		No	2
2.05	During this visit, did you give a blood sample?	Yes	1
		No	2
2.06	During this visit, did the provider palpate your tummy?	Yes	1
		No	2
2.07	During this visit, was your uterine height	Yes	1
	measured?	No	2
2.08	During this visit, did a health worker give you iron pills, folic acid or iron with folic acid, or give you a prescription for them?	Yes	1
		No	2
2.09	Was an ultrasound done?	Yes	1
		No	2
2.10	Were medicines dispensed to you?	Yes	1
		No	2

(3) **COUNSELLING**

3.01	During this visit, did a health worker give you advice on your diet?	Yes	1
		No	2
3.02	During this visit, did a health worker give you advice on how to use your medications?	Yes	1
		No	2
3.03	During this visit or previous visits, has a health worker talked with you about any signs of complications (danger signs) that should warn you of problems with the pregnancy?	Yes, during this visit	1
		Yes, previous visit	2
		No	3
3.04	During this visit, did a health worker talk with you about using family planning after the birth of your baby?	Yes	1
		No	2
3.05	During this visit or previous visits, has a provider given you advice on the importance of exclusively breastfeeding	Yes, during this visit	1
		Yes, previous visit	2
		No	3
3.06	During this visit or previous visits, did the provider talk to you about where you plan to	Yes, during this visit	1
		Yes, previous visit	2

	deliver your baby?	No	3

(4)	**TRAVEL AND EXPENDITURE**		
4.01	How far is your household from this health facility?	Kilometers	

(5) **PATIENT SATISFACTION**	Agree	3
	Neutral	2
	Disagree	1

The health staff are courteous and respectful

The health workers did a good job of explaining your health condition.

It is easy to get medicine that health workers prescribe.

The amount of time you spent waiting to be seen by a health provider was reasonable.

You had enough privacy during your visit.

The health worker spent a sufficient amount of time with you

The hours the facility is open are adequate to meet your needs

The health workers in this facility are extremely thorough and careful.

You trust in the skills and abilities of the health workers of this facility.

You completely trust the health worker's decisions about medical treatments in this facility.

The health workers in this facility are very friendly and approachable.

The health workers in this facility are easy to make contact with.

The health workers in this facility care about your health just as much or more than you do.

Thank you
