# Supplementary material for: Assessment of Pregnant Women’s Satisfaction with Model of Care Initiative: Antenatal Care Service at Primary Health Care in Cluster One in Riyadh, Saudi Arabia
Source: Healthcare (Basel). 2022 Jan 13;10(1):151. doi: 10.3390/healthcare10010151 (PMC8775455; doi:10.3390/healthcare10010151)
Supplement: Supplementary file 1 [file healthcare-10-00151-s001.zip › healthcare-1511944-supplementary Table S1.pdf]

Supplementary Material

Supplementary Table S1. The sociodemographic characteristics of the participants (n=646)

Variables	Categories	No.	%
Age groups (years)	≤25 years	161	24.9
	26-35 years	352	54.5
	≥36 years	133	20.6
Education	Basic	115	17.8
	Secondary	302	46.7
	University	229	35.4
Monthly income	≤4900 SR	215	33.3
	5000-7000 SR	255	39.5
	>7000 SR	176	27.2
Number of Pregnancies	One pregnancy	175	27.1
	2-3 pregnancies	328	50.8
	≥4 pregnancies	143	22.1
First visit to the PHC	yes	253	39.2
	no	393	60.8
